# Grafting glycoprotein-derived oligosaccharides structures onto non-glycosylated polypeptides

**DOI:** 10.17912/micropub.biology.001314

**Published:** 2024-10-30

**Authors:** Danya Medina-Carrasco, Lisandra García de Castro Cuspineda, Luis Gabriel González-Lodeiro, Satomy Pousa, Miladys Limonta, Vivian Huerta Galindo

**Affiliations:** 1 Centro de Ingeniería Genética y Biotecnología, Havana, Havana, Cuba

## Abstract

Properties of recombinant glycoproteins can be altered by the addition of oligosaccharide structures specific to the cells used for its heterologous expression. A methodology was implemented to obtain a glycopeptide preparation useful to elucidate the role of carbohydrates in the immunogenicity and antigenicity of glycoproteins. It consists on the digestion of the protein, followed by selective capture of the oligosaccharides bound to di-/tripeptides, and their grafting onto a non-glycosylated receptor protein by chemical crosslinking. Glycopeptides derived from C-RBD-H6 PP protein, the active ingredient of the Abdala vaccine were efficiently grafted onto a non-glycosylated protein as evidenced by western blotting.

**Figure 1. Grafting C-RBD-H6 PP glycopeptides onto DIIIE1 protein:**
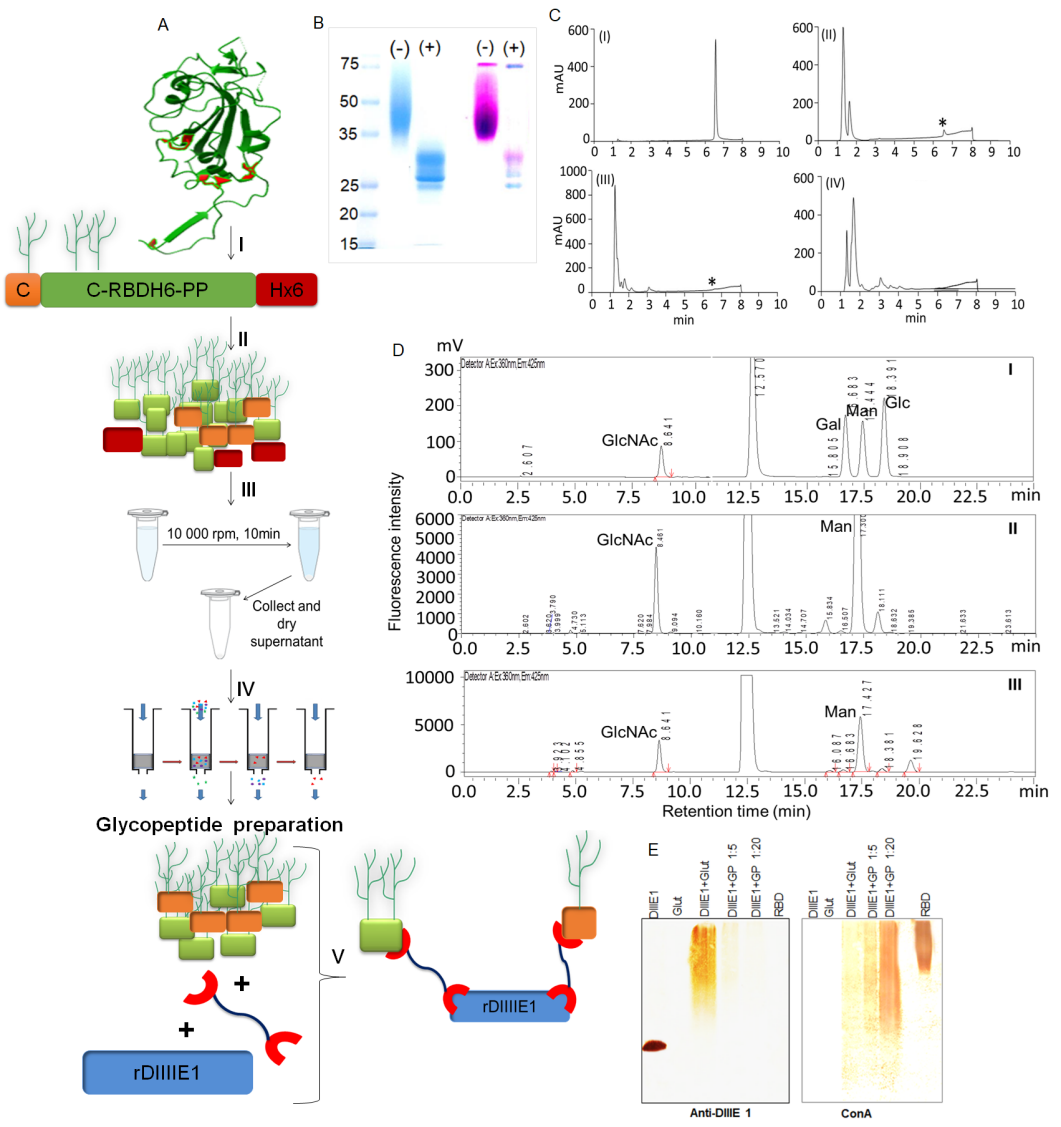
(A) Experimental strategy followed to obtain the glycopeptide preparation and grafting to DIIIE1 protein: (I) the reduction of the disulfide bridges and S-alkylation of cysteine ​​residues, (II) sequential digestion with two low-specificity proteases, the enzymes Pronase E and proteinase K, (III) alcoholic precipitation of intact protein residues, (IV) capture of oligosaccharide structures with a graphite column and (V) grafting the glycopeptides onto DIIIE1 protein. (B) SDS-PAGE analysis of C-RBD-H6 PP protein before (-) and after (+) treatment with PNGase F. The result of Coomasie Blue staining and Schiff staining of the same gel is presented. (C) RP-HPLC analysis of (I) intact C-RBD-H6 PP, (II) product of digestion with Pronase E, (III) product of successive digestion with proteinase K and (IV) elution of the graphite column chromatography. (D) Analysis of monosaccharide content by derivatization with 2-aminobenzoic acid and separation in chromatography on HPLC. The elution profiles of (I) standard monosaccharide mixture, (II) monosaccharide content of the C-RBD-H6 PP protein, and (III) monosaccharide content of the glycopeptide preparation are shown. (E) Western blotting analysis of the transfer of C-RBD-H6 PP glycopeptides to the non-glycosylated recombinant protein DIIIE1. Detection of DIIIE1 with anti-DIIIE1 serum, and with an anti-concanavalin A: horseradish peroxidase (ConA) conjugate, as indicated. GP. Glycopeptide preparation. Glut. Glutaraldehyde. RBD. C-RBD-H6 PP protein. Lanes that show the product of the crosslinking reaction are labelled DIIIE1+GP using two different proportions C-RBD-H6 PP:GP, 1:5 and 1:20, as indicated in the figure.

## Description

Glycosylation is one of the most frequent post-translational modifications in proteins from eukaryotic cells (Hu, Guo, & Li, 2006) . The glycans that modify glycoproteins have been associated with functions such as contributing to folding, stability, and solubility, mediating the reactivity of the immune system, cell adhesion and differentiation (Lembo, Molinaro, De Castro, Berti, & Biagini, 2024) . The development of methodologies to elucidate the contribution of oligosaccharides to the functional properties of glycoproteins is of major importance for research groups working in protein characterization.

An et al., 2003 described an approach to obtain glycans linked to small peptides using extensive proteolytic digestion with Pronase E (An, Peavy, Hedrick, & Lebrilla, 2003) . Pronase E is can hydrolyze a large number of protein peptide bonds with the subsequent liberation of 70 to 90% of free amino acids. In glycoproteins, the presence of oligosaccharide chains can limit proteolytic cleavage at neighboring amino acids and lead to the formation of di- or tripeptides carrying the oligosaccharide chains (An et al., 2003) . In this work we apply a similar experimental strategy but, glycoprotein derived glycopeptides are transplanted to polypeptides of unrelated sequence. Different from glycoside grafting strategies based on chemical synthesis that use a controlled polymerization of glycosylated monomers over a polypeptide (Clauss et al., 2021) , the experimental approach implemented in this work preserves the entire diversity in the composition and structures of the glycans present in the protein of interest in a conjugate with a selected polypeptide.

The experimental strategy is based on the total proteolytic digestion of the protein with non-specific proteases, followed by the selective isolation of the oligosaccharide structures on a graphite column ( Figure 1A ). To ensure better access of proteases to all cleavage sites, the protein is first subjected to reduction of disulfide bonds and S-alkylation of cysteine residues (Step I, Figure 1A ). Next, a sequential proteolysis is carried out with Pronase E and proteinase K proteases (Step II, Figure 1A ). A total digestion of the protein guarantees that epitopes involving the primary structure of the protein are eliminated, which allows to analyze the contribution of oligosaccharide structures. An alcoholic precipitation (Step III, Figure 1A ) is used to separate the enzymes and large protein fragments from the released glycopeptides. The oligosaccharide chains that remained in solution are selectively concentrated in a graphite column (Step IV, Figure 1A ), which also eliminate small non-glycosylated polypeptide fragments. Finally, the glycopeptide preparation is grafted to another polypeptide using a chemical cross-linking reagent (Step V, Figure 1A ). The glycopeptide conjugate can be used to study the effect of oligosaccharide structures on functional characteristics of glycoproteins such as immunogenicity, antigenicity, or the ability to mediate interactions with other biomolecules, independently of the polypeptide structure.

The yeast P. pastoris is a very useful expression system to obtain proteins for therapeutic purposes due to its low production cost, rapid growth, and the potential to introduce post-translational modifications such as disulfide bonds and N- and O-glycosylation in proteins (Cereghino & Cregg, 2000) . To evaluate the feasibility of the proposed methodology, the protein C-RBD-H6 PP was used as a model of a hyperglycosylated protein expressed in P. pastoris (Vervecken et al., 2004) . This protein constitutes the active ingredient of the Abdala vaccine, used in the prevention of Covid-19 (Hernández-Bernal et al., 2022; Limonta-Fernández et al., 2022). Like any vaccine preparation for use in humans, it is of major interest to obtain a detailed characterization of structural determinants of its functional properties.

The genetic construct of C-RBD-H6 PP protein comprises the receptor binding domain (RBD, Asn _331_ -Lys _529_ ) of the SARS-CoV-2 S protein (Uniprot ID P0DTC2). In this sequence fragment the two N-glycosylation sites of the RBD are modifying residues Asn _331_ and Asn _343_ (Grant, Montgomery, Ito, & Woods, 2020; Wang, Wang, & Zhuang, 2020) ; the RBD domain is flanked by flexible linkers rich in Glycine and Serine, which are used as connectors in the fusion protein (Van Rosmalen, Krom, & Merkx, 2017). These sequence segments create new potential N- and O-glycosylation sites (Limonta-Fernández et al., 2022).

In the SDS-PAGE analysis, the C-RBD-H6 PP protein migrates as a dispersed band between 35 and 75 kDa ( Figure 1B ), which is higher than the expected molecular mass (26 kDa) of the protein (Limonta-Fernández et al., 2022). Treatment with basic Fuchsin ( Figure 1B ) demonstrates the presence of glycosylated species throughout the distribution of the C-RBD-H6 PP band. When treating the protein with the glycosidase PNGase F, the protein migrates at a molecular mass close to 26 kDa, confirming that the increase in size is mainly due to the presence of N-glycosylation. Basic Fuchsin staining of the deglycosylated protein also shows the presence of protein bands close to 26 kDa that preserve associated oligosaccharides, and suggest the presence of O-glycosylation in this protein.

To evaluate the efficiency of the proteolytic digestion, the profile of the intact protein in RP-HPLC analysis was compared with the profiles after each protease treatment ( Figure 1C, sections I, II and III). The digestion conditions guaranteed the total disappearance of the peak eluting at 6.5 minutes that corresponds to intact C-RBD-H6 PP ( Figure 1C, I ). At the same time, it appears a peak with an elution time of 1 minute, indicating the presence of structures of very low hydrophobicity, as expected for glycosylated polypeptides. The presence of glycan structures in this peak was confirmed by quantitation of the carbohydrate content by the anthrone-sulfuric acid assay. After the alcoholic precipitation and concentration steps, the sample was analyzed in RP-HPLC, showing good recovery of the glycans ( Figure 1C, section IV). To characterize the glycopeptide preparation, the monosaccharide composition of C-RBD-H6 PP protein ( Figure 1D, section II), was compared with monosaccharide content of the glycopeptide preparation ( Figure 1D, section III). Both samples have a similar chromatographic profile that evidenced the presence of N-acetyl glucosamine (GlcNAc) and mannose (Man), as major species. Together, results of quantitative and qualitative characterization evidence that the methodology used allows an efficient recovery of the glycans present in the intact protein.

The glycopeptide preparation obtained was grafted onto DIIIE1 protein by chemical crosslinking. DIIIE1 protein comprises the structural domain III (Met _289_ -Gly _399_ ), of the envelope protein of dengue virus, serotype 1 (Huerta et al., 2014) . DIIIE1 protein is obtained by heterologous expression in E. coli and does not present potential glycosylation sites in its amino acid sequence. The homobifunctional crosslinker glutaraldehyde was used to covalently bind glycopeptides to DIIIE1 protein. Two conditions were used in the crosslinking reaction, varying the excess of glycopeptides with respect to the mass of the receptor protein (5:1 and 20:1, Figure 1E ). The result of the grafting was evaluated in western blotting ( Figure 1E ). DIIIE1 protein is recognized by the Concanavalin A conjugate only after the crosslinking reaction. The degree of modification of DIIIE1 raise with the increase in the proportion of glycopeptides in the reaction, which can be seen from the increase in the recognition signal with the Concanavalin A conjugate. At the same time, as the modification of DIIIE1 protein increases with the oligosaccharide structures, it decreases recognition with the anti-DIIIE1 serum ( Figure 1E ) which is probably related to steric hindrance of the oligosaccharide chains in the binding of the antibodies and modification of key residues of antibody epitopes. One caveat of the protocol, as presented in this example, is that the crosslinking stoichiometry of glutaraldehyde is difficult to control, and it can cause intramolecular or intermolecular crosslinking of DIIIE1 molecules, as shown in the western image ( Figure 1E ). Nonetheless, our current preliminary results evidence that glycopeptide grafting may efficiently work using other crosslinkers like homobifunctional imidoesters which can be used in more stoichiometrically controlled reactions for the modification of primary amines in proteins. The conjugate shown in Figure 3E can be used as antigen in immunization schemes or immunoassays to investigate the role of oligosaccharide structures in vaccine immunogenicity and antigenicity.

## Methods

**Proteins**

A purified preparation of C-RBD-H6 PP protein was obtained from an experimental batch from the Direction of Downstream Process Development of the Center for Genetic Engineering and Biotechnology of Havana, Cuba. DIIIE1 protein was obtained by heterologous expression in *E. coli* and purified as previously described (Huerta et al., 2014) .

**Protein denaturation**

Ten milligrams of C-RBD-H6 PP protein were reduced for 2 h at 37°C with 10 mM dithiothreitol 100 mM Tris pH 8.0, 2 M urea. Free cysteine residues were alkylated with 20 mM iodoacetamide for 20 minutes at 25°C. At the end of the reaction, the protein preparation was desalted by gel filtration using a PD10 column (GE Healthcare, USA) equilibrated with Tris 20 mM pH 8.0, CaCl _2_ 10 mM. Finally, protein was incubated at 80°C for 1h and stored at -20°C until used.

**Proteolytic digestion**

Denature protein was treated sequentially with Pronase E and Proteinase K in Tris 20 mM pH 8.0, CaCl _2_ 10 mM. In each reaction the enzyme was added in a protease:protein ratio (w/w) of 5:1 and the reaction was incubated with for 48h at 37°C.

**Analytical chromatography in Reversed-phase high-performance liquid chromatography (RP-HPLC)**

An analytical column Chromolith Performance RP-18e (Merck, Germany) was used for RP-HPLC analysis connected to an AKTA Pure chromatographic system (GE Healthcare, USA) with online detection at 226 nm. Protein samples were diluted with 0.1% trifluoroacetic acid and loaded to the column at a flow rate of 0.5 ml /min. Column was washed at 5 ml/min with 0.1% TFA until absorbance return to baseline. The sample was eluted with a step gradient from 0.1% TFA to 30% acetonitrile in 4 min, 30-60% acetonitrile in 2 min and 60-100% acetonitrile in 1 min. All acetonitrile solutions contained 0.1% TFA. 

**Alcoholic precipitation**

The peptide mixture resulting of the proteolytic digestion was mixed with of cold absolute ethanol in a ratio 1:3 (v/v) and incubated for 1 h at -20°C. Next, the sample was centrifuged at 10,000 rpm for 10 minutes at 4°C. The supernatant was collected and dried in a rotary evaporator.

**Quantitation of carbohydrate content**

The quantitation of the carbohydrate content was performed by the anthrone-sulfuric acid assay (Leyva et al., 2008) . The anthrone reagent (0.1% anthrone in sulfuric acid) was prepared right before analysis. Hundred and fifty microliters of anthrone reagent was added to 50 μL of each sample. Plates were then placed for 10 min at 4°C and successively at 100°C for 20 min. After cooling for 20 min at room temperature, results were registered by reading absorbance at 620 nm. The standard curve was based on methyl-α-D-mannopyranoside (Sigma, USA).

**Purification of oligosaccharides on a graphite column**

The purification of the oligosaccharides was carried out with a Alltech® Carbograph cartridge (0.8 cm × 0.8 cm; Alltech, The Netherlands), following the procedure recommended by the provider. Briefly, the column was equilibrated with 3 ml of 50% acetonitrile, 0.1% TFA followed by 6 ml of 5% acetonitrile, 0.1% TFA. The sample was dissolved in 100 µl of water and loaded to the column at gravity flow. Next, the column was washed with 3 ml of water and 3 ml of 5% acetonitrile, 0.1% TFA. Bound oligosaccharides were eluted with 4 applications of 0.5 ml of 50% acetonitrile, 0.1% TFA. The eluates were pooled and the sample dried in a rotary evaporator. 

**Determination of monosaccharide content**

A Ludger Tag® 2-AA Monosacharide Release and Labelling kit was used to determine the monosacharide content. Two separate aliquots of either C-RBD-H6 PP or the glycopeptide preparation were hydrolyzed with 2M TFA or HCL for 3 hours at 100°C. Hydrolyzed samples were dried in a rotary evaporator and reducing ends were labeled with the fluorophore 2-aminobenzoic acid: sodium cyanoborohydride (1:1) for 1 h at 80°C. Labelled monosaccharides were separated on HPLC using a LudgerSepR1 column equilibrated with 0.2% butylamine, 0.5% orthophosphoric acid, 1% tetrahydrofuran (BPT solvent), and eluted with the recommended step gradient with increasing concentrations of 50%: acetonitrile: 50% BPT. The column was operated at 0.8 ml/min and 30°C. The retention time was compared with that of a monosaccharide standard profile containing GlcNAc, Gal, Man and Glu.

**Crosslinking reaction**

Protein samples and glycopeptide preparation were dissolved in PBS and Glutaraldehyde solution was added to a final concentration of 0.5%. The reaction mixture was incubated for 20 min at 25°C. To stop the reaction, Gly 1M pH 8.0 to obtain a 0.2M of final concentration was added. 

**Denaturing polyacrylamide gel electrophoresis (SDS-PAGE)**

Polyacrylamide gels were prepared according to the standard conditions described by Laemmli (Laemmli, 1970) and protein bands were visualized by Coomasie blue staining. All incubations for protein and carbohydrate staining were performed at 25°C with constant shaking. After recording the image of the coomasie blue stained gel, the protein-associated carbohydrates were detected by periodic acid–Schiff´s staining (Moravec & Mares, 2017) . To this end, coomasie blue staining was removed by incubation of the gel in 10% acetic acid, 40% methanol until proteins bands were not visible. Then, sodium periodate solution 5% in acetic acid was added and the gel was incubated for 1 h followed by incubation for 10 min with sodium metabisulfite solution 5% acetic acid. Carbohydrate structures were developed by the addition of a solution of basic Fuchsin.

**Western blotting analysis**

After SDS-PAGE separation, protein bands were transferred to nitrocellulose at 300 mA for 2 h using 25 mM Tris pH 8.3, 192 mM glycine, 20% (v/v) methanol, (Towbin, Staehelin, & Gordon, 1979) . The membranes were blocked with 3% bovine serum albumin in PBS with 0.05% Tween 20 (PBST), pH 7.4 for 1 h at 37 °C. After three washes of 10 min each with PBST, the membranes were incubated for 1 h at 37°C, with an anti-DIIIE1 polyclonal preparation obtained in rabbits or with Concanavalin A conjugated to horseradish peroxidase. For the detection of anti-DIIIE1 antibodies, an anti-rabbit IgG specific antibody conjugated to horseradish peroxidase was applied at the dilution recommended by the provider. Subsequently, the membranes were washed and color developed using the peroxidase substrate mixture 1 mg/ml 3,3'-diaminobenzidine at 1 mg/ml, 0.03% H _2_ O _2_ in PBST.
